# Preoperative Ocular Biometric Parameters as Predictors of Intraocular Pressure Reduction After Phacoemulsification Cataract Surgery in Non-Glaucomatous Eyes †

**DOI:** 10.3390/life15030381

**Published:** 2025-02-28

**Authors:** Feyzahan Uzun, Hüseyin Findik, Muhammet Kaim

**Affiliations:** Department of Ophthalmology, School of Medicine, Recep Tayyip Erdogan University, 53100 Rize, Turkey; huseyin.findik@erdogan.edu.tr (H.F.); muhammet.kaim@erdogan.edu.tr (M.K.)

**Keywords:** anterior chamber depth, cataract surgery, intraocular pressure, lens thickness

## Abstract

Purpose: This study aimed to evaluate the relationship between preoperative ocular biometric parameters and intraocular pressure (IOP) reduction after phacoemulsification in non-glaucomatous eyes. Methods: The charts of non-glaucomatous patients who underwent phacoemulsification and lens implantation were retrospectively reviewed. IOP was measured preoperatively and 3 months after surgery. The change in IOP and its relation to ocular biometric parameters, including anterior chamber depth (ACD), lens thickness (LT), axial length (AL), and central corneal thickness (CCT), measured preoperatively by LENSTAR LS 900 (Haag-Streit, Inc., Koeniz, Switzerland), were evaluated. The impact of each parameter on the alteration in IOP was assessed using a regression model. Results: The analysis included 171 eyes of 145 patients, with a mean age of 68.7 ± 11 years. The average IOP reduction following cataract surgery was 3.0 ± 2.9 mmHg, decreasing from a preoperative mean of 16.3 ± 2.8 mmHg. The amount of IOP reduction showed statistically significant correlations with preoperative ocular parameters. Eyes with shallower anterior chambers demonstrated a greater IOP reduction (r = −0.307, *p* = 0.034), as did those with thicker lenses (r = 0.383, *p* = 0.026). Multivariate regression analysis further confirmed that higher preoperative IOP, shallower ACD, and thicker lenses were independently associated with greater postoperative IOP decreases (*p* < 0.001). AL and CCT did not exhibit significant associations with IOP reduction. Conclusions: The amount of IOP reduction is significantly greater in eyes with higher preoperative IOP, thicker lenses, and shallower anterior chamber after cataract surgery in non-glaucomatous eyes.

## 1. Introduction

In recent years, increasing attention has been directed toward the effect of cataract surgery on intraocular pressure (IOP), a key modifiable risk factor in glaucoma management. Numerous studies have demonstrated that cataract surgery leads to a reduction in IOP in both glaucomatous and non-glaucomatous eyes, though the degree of this reduction varies [1]. In angle-closure glaucoma (ACG), cataract extraction is well established as an effective intervention, often leading to substantial and sustained IOP reduction by deepening the anterior chamber and widening the drainage angle [2]. Notably, the recent EAGLE trial highlighted the superiority of clear lens extraction over laser peripheral iridotomy for treating ACG, showing improvements in IOP, quality of life, and treatment costs [3]. In contrast, the effect of cataract surgery on IOP in open-angle eyes—whether glaucomatous or non-glaucomatous—remains less predictable, and the underlying mechanisms are not yet fully understood [1].

Several hypotheses have been proposed to explain IOP reduction after cataract surgery in open-angle eyes. These include mechanical changes that enhance trabecular outflow, low-grade inflammation reducing aqueous production, and alterations in Schlemm’s canal patency and trabecular meshwork structure—similar to the effects of selective laser trabeculoplasty [4]. Despite these theoretical mechanisms, there is still no consensus on the key ocular biometric factors that predict the magnitude of postoperative IOP reduction in non-glaucomatous eyes. Although previous studies have explored various preoperative parameters such as anterior chamber depth, lens thickness, and angle configuration, the predictive value of these factors remains inconsistent across different populations and study designs [5,6].

With the advent of advanced imaging technologies such as anterior segment optical coherence tomography (OCT) and Scheimpflug imaging, a more detailed assessment of anterior segment anatomy is now possible. These tools offer an opportunity to refine our understanding of the relationship between preoperative ocular biometry and IOP outcomes following cataract surgery. However, comprehensive data on how specific biometric parameters influence IOP reduction in non-glaucomatous eyes are still lacking.

In this study, we aim to address this gap by systematically evaluating the associations between preoperative ocular biometric measurements and postoperative IOP reduction in non-glaucomatous eyes. By leveraging high-resolution imaging techniques, we seek to identify reliable anatomical predictors of IOP change, which could aid in personalized surgical planning and enhance preoperative counseling for patients undergoing cataract surgery. Unlike previous studies that primarily focused on glaucomatous eyes or broader patient populations, our study specifically investigates non-glaucomatous eyes, providing new insights into the mechanisms underlying IOP reduction in this subset of patients.

## 2. Materials and Methods

In this study, the charts of non-glaucomatous patients who underwent clear corneal phacoemulsification and foldable intraocular lens implantation were retrospectively reviewed. All participants were informed about the surgical procedures, and written informed consent was obtained prior to the surgery. This study adhered to the principles of the Declaration of Helsinki, and the protocol received approval from the Recep Tayyip Erdogan University Ethics Committee.

The inclusion criteria for patients undergoing cataract surgery were as follows: age over 18 years, the presence of a visually significant cataract with a best-corrected visual acuity worse than 20/40, an IOP ≤ 20 mmHg, and open anterior chamber angles (Schaffer grade III or IV) as determined by gonioscopy. Preoperatively, all patients underwent a detailed ophthalmological examination, including assessment of best-corrected visual acuity, biomicroscopy, gonioscopy to confirm open angles, Goldmann applanation tonometry, and fundus examination. The IOP measurements were conducted one day prior to surgery between 1:00 PM and 4:00 PM. For analysis, the average of three consecutive measurements was used. Ocular biometric parameters, including axial length (AL), anterior chamber depth (ACD), lens thickness (LT), and central corneal thickness (CCT) were measured using non-contact ocular biometry, Lenstar LS 900 (Haag-Streit, Inc., Koeniz, Switzerland). Five readings were taken for each eye by an experienced ophthalmic technician, after excluding the highest and lowest values, and the mean of the remaining three readings was used for analysis.

To minimize potential confounding factors, patients with a history of intraocular surgery, coexisting ocular pathologies (e.g., uveitis, diabetic retinopathy, retinal vascular occlusion), or any form of glaucoma were excluded from the study. Additionally, patients using systemic or topical medications known to influence IOP were not included.

The surgical procedure utilized was the stop-and-chop phacoemulsification technique, performed through a 2.8 mm superior clear corneal incision under topical anesthesia. A foldable intraocular lens (IOL; AcrySof IQ SN60WF, Alcon Laboratories Inc., Fort Worth, TX, USA) was implanted in the capsular bag. Patients with intraoperative complications (e.g., posterior capsule rupture, zonular instability) were excluded from the study. Postoperatively, all patients received a standardized treatment regimen consisting of 0.5% moxifloxacin and 1% prednisolone acetate eye drops. The drops were administered five times daily for the first week and were then tapered and discontinued over a three-week period. Follow-up evaluations were conducted at 1 day, 1 week, 1 month, and 3 months postoperatively. These assessments included visual acuity testing, IOP measurement, and a comprehensive slit-lamp examination. To ensure consistency in IOP measurements and reduce variability, postoperative IOP was measured at the same time of day as the preoperative measurements (between 1:00 PM and 4:00 PM).

### Statistical Analysis

All analyses were conducted using SPSS for Windows version 27.0 (IBM Corp., Armonk, NY, USA), a comprehensive software package for statistical analysis. Descriptive statistics, including the calculation of mean and standard deviation, were used to summarize the data. To compare IOP values before and after surgery, the Wilcoxon signed-rank test was applied. To explore the relationships between different biometric parameters and the change in IOP, the Pearson correlation test was utilized. Multivariate regression analysis was performed to assess the influence of preoperative parameters on the percentage of change in IOP post-surgery. This analysis provides insight into how each parameter affects the outcome variable, allowing for a more detailed understanding of the factors contributing to IOP changes. The significance level for all statistical tests was set at 0.05, ensuring that the results were statistically robust. An a priori power analysis was conducted using G*Power version 3.1.9.7 to determine the minimum sample size required to test the study hypothesis. The results indicated that a total sample size of minimum N = 147 would be required to achieve 95% power for detecting a large effect (0.3), with a significance criterion of α = 0.05.

## 3. Results

### 3.1. Patient Characteristics and Preoperative Ocular Biometric Parameters

A total of 171 eyes from 145 patients (65 females, 80 males; mean age: 68.7 ± 11 years) were included in the study. Table 1 summarizes the baseline characteristics and ocular biometric parameters. The mean ACD was 2.63 ± 0.46 mm, while the mean LT and AL were 4.40 ± 0.38 mm and 23.2 ± 0.91 mm, respectively. The mean CCT was 534 ± 50.1 μm.

### 3.2. Changes in IOP Following Cataract Surgery

Table 2 presents the preoperative and postoperative IOP measurements. The mean preoperative IOP was 16.3 ± 2.8 mmHg (range: 9–25 mmHg), which decreased significantly to a mean postoperative IOP of 14.4 ± 3.1 mmHg (range: 8–20 mmHg) (*p* < 0.001). The absolute change in IOP averaged −3.0 ± 2.9 mmHg, with a range from −12.0 to +1.0 mmHg. The mean percentage change in IOP was −15.4 ± 18.7%, ranging from −50.0% to +7.1%.

### 3.3. Correlation Analyses of Predictors for IOP Reduction

**Figure 1:**
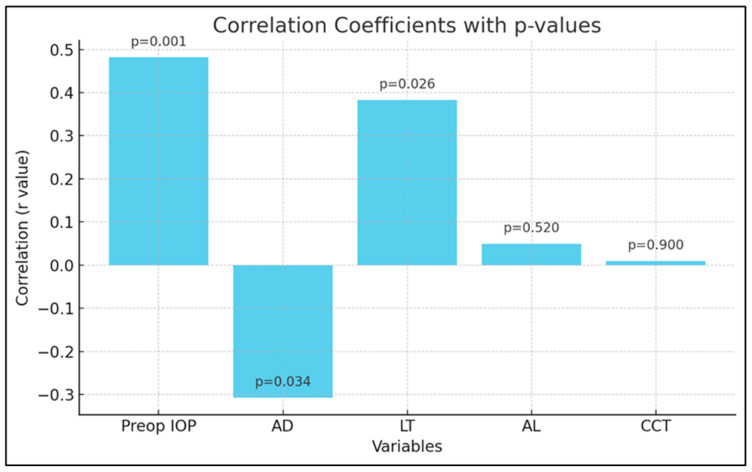
Correlation coefficients for predictors of percent IOP change after cataract surgery.

Correlation analyses (Table 3, Figure 1) indicated that a higher preoperative IOP was significantly associated with a greater percentage reduction in IOP (r = 0.482, *p* < 0.001). Additionally, both ACD and LT showed significant correlations with the percentage of IOP change. ACD was inversely correlated with IOP reduction (r = −0.307, *p* = 0.034), suggesting that shallower anterior chambers were associated with greater IOP decreases postoperatively. Conversely, LT was positively correlated with IOP reduction (r = 0.383, *p* = 0.026). AL and CCT did not show significant correlations with IOP change (*p* = 0.52 and *p* = 0.9, respectively).

### 3.4. Regression Analysis of Preoperative Predictors for IOP Reduction

Multivariate regression analysis (Table 4) identified preoperative IOP, ACD, and LT as significant predictors of the percentage of IOP reduction. Preoperative IOP was a strong predictor, with a coefficient of 0.354 (95% CI: 0.243 to 0.465, *p* < 0.001). ACD was negatively associated with IOP change, with a coefficient of −0.853 (95% CI: −1.492 to −0.214, *p* = 0.009), highlighting that smaller ACD predicts a greater reduction in IOP. LT also showed a positive association with IOP change (coefficient: 1.48, *p* < 0.001), indicating that increased LT contributes to a higher percentage of IOP reduction following surgery.

## 4. Discussion

This study examined the relationship between preoperative ocular biometric parameters and IOP reduction following cataract surgery in non-glaucomatous eyes. The findings confirm that cataract surgery results in a measurable reduction in IOP, even in eyes without glaucoma. Moreover, the results emphasize the predictive value of specific preoperative ocular parameters in determining the extent of this reduction. Specifically, greater IOP reduction was observed in eyes with higher preoperative IOP, thicker lenses, and shallower anterior chambers.

Recent studies have consistently demonstrated that cataract surgery lowers IOP in non-glaucomatous eyes. Researchers have reported a mild-to-moderate reduction in IOP postoperatively, averaging 1–3 mmHg [7]. One study confirmed that this reduction persists for at least two years in patients without pre-existing glaucoma [8]. The underlying mechanism is thought to involve enhanced aqueous outflow due to anterior chamber deepening and angle widening following cataract removal. Notably, the decrease in IOP is more pronounced in eyes with higher preoperative IOP, suggesting a potential therapeutic benefit for patients with marginally elevated IOP [9]. Additionally, cataract surgery has been shown to be particularly effective in older adults with mature cataracts [10]. These findings underscore the potential role of cataract surgery in mitigating glaucoma risk in patients with borderline IOP. Consistent with previous research, our study observed a mild-to-moderate IOP reduction postoperatively, likely attributable to anatomical changes that facilitate aqueous drainage.

Cataract surgery is known to reduce postoperative IOP, and recent studies have aimed to identify predictive factors for this decrease. Advanced imaging tools, such as anterior segment OCT and Scheimpflug imaging, have enabled precise quantification of anatomical changes, confirming their role in IOP modulation after surgery. Several preoperative factors have been identified as predictors of IOP reduction following cataract surgery. A higher preoperative IOP is one of the most consistent predictors, with greater postoperative reductions observed in eyes with elevated baseline IOP [6]. Shallow ACD and increased lens vault, as assessed by anterior segment imaging techniques such as anterior segment OCT and Scheimpflug imaging, have also been associated with a more pronounced IOP decrease, likely due to the relief of crowding in the anterior chamber after lens extraction [7]. Sarkar et al. reported a significant reduction in IOP following cataract surgery in non-glaucomatous eyes, identifying preoperative IOP and lens vault as the most important predictors. They speculated that replacing the thick cataractous lens with a thinner intraocular lens led to changes in anterior segment parameters, resulting in anterior chamber deepening and angle widening [11]. In another study, researchers investigated potential predictors of IOP change with cataract surgery in patients with pseudoexfoliative glaucoma and without glaucoma. Preoperative IOP and pressure-to-depth ratio (PD) [PD ratio = preoperative IOP/preoperative ACD] were found to be predictors in both groups [12]. DeVience et al. examined intraoperative factors alongside biometric parameters and identified higher preoperative IOP, a more anterior relative lens position, and longer phaco time as significant predictors of greater IOP reduction following cataract surgery [13]. Additionally, smaller anterior chamber angles and higher lens thickness have been linked to greater postoperative IOP reduction, particularly in patients with anatomically narrow angles or primary angle-closure suspects [14]. Other factors, such as axial length, corneal biomechanics, and baseline outflow facility, may also influence the extent of IOP reduction [15]. Our findings suggest that patients with higher preoperative IOP, thicker lenses, and shallower anterior chambers are more likely to experience a significant reduction in IOP following cataract surgery. Understanding these preoperative parameters is essential for predicting surgical outcomes and optimizing patient selection, particularly in individuals with ocular hypertension and glaucoma. Biometric factors related to angle configuration and lens position have been identified as critical determinants of postoperative success. This highlights the potential role of preoperative biometric evaluation in guiding surgical decision-making, particularly in individuals with borderline elevated IOP or anatomically crowded anterior segments. Although lowering IOP is not a primary goal in non-glaucomatous patients, certain individuals—particularly those with ocular hypertension or at risk for glaucoma—may benefit from closer postoperative monitoring. Identifying patients who are more likely to experience a significant reduction in IOP could influence treatment strategies, such as considering earlier cataract surgery in selected cases to prevent IOP-related complications.

Although cataract surgery leads to a modest and sustained reduction in IOP in both POAG and ACG patients and patients without glaucoma, its hypotensive effect is more pronounced in ACG due to the anatomical widening of the anterior chamber following lens extraction [1,16]. Ismi et al. compared the effect of cataract surgery on IOP in patients with and without glaucoma and observed that IOP decreased in all groups, but the change was significant in ACG group [17]. Hayashi et al. reported that cataract surgery resulted in an increase in both the width and depth of the anterior chamber angle, leading to a significant reduction in IOP in patients with ACG [18]. A study reported that phacoemulsification with foldable intraocular lens implantation significantly deepens the anterior chamber, widens the drainage angle, and reduces IOP, with a greater reduction observed in narrow-angle eyes (18%) compared to open-angle eyes (10%), proportional to the increase in angle width [14]. The removal of the crystalline lens through phacoemulsification deepens the anterior chamber, widens the drainage angle, and reduces resistance to aqueous humor outflow, thereby lowering IOP, particularly in anatomically predisposed eyes [16]. While the mechanisms underlying IOP reduction in open-angle configurations remain less clear, potential contributors include enhanced trabecular meshwork function due to mechanical remodeling and low-grade inflammatory responses [19,20].

This study has several limitations, including a relatively small sample size and a follow-up duration of 3 months. Extending this study beyond this period with a larger sample size would allow for a more comprehensive observation of postoperative IOP changes and provide a better understanding of the long-term stability of IOP after cataract surgery. Additionally, while we focused on non-glaucomatous eyes, future research should explore how these findings compare to outcomes in eyes with different types of glaucoma. Furthermore, demographic variations, such as age distribution and systemic comorbidities, were not specifically analyzed in this study, which may influence postoperative outcomes. Future studies with diverse patient populations will help to generalize these findings more effectively.

In conclusion, our study highlights the critical role of preoperative ocular biometrics in understanding and predicting the IOP-lowering effects of cataract surgery in non-glaucomatous eyes. These findings contribute to the growing body of evidence supporting the multifaceted benefits of cataract surgery, not only for visual rehabilitation but also for IOP management. By focusing on individuals with high-risk biometric profiles, cataract surgery may serve as a preventive intervention for IOP-related complications in aging populations. Moreover, our results provide a foundation for future investigations into optimizing surgical outcomes through precision medicine approaches, which could enhance both the efficacy and safety of cataract surgery in diverse patient populations.

## Figures and Tables

**Table 1:** Patient characteristics and preoperative ocular biometric parameters.

Parameter	Mean ± SD
Age (years) (range)	68.7 ± 11 (41–82)
Gender (n) (female/male)	65/80 (44.8–55.2%)
ACD (mm)	2.63 ± 0.46
LT (mm)	4.40 ± 0.38
AL (mm)	23.2 ± 0.91
CCT (µm)	534 ± 50.1

ACD: anterior chamber depth, LT: lens thickness, AL: axial length, CCT: central corneal thickness, SD: standard deviation.

**Table 2:** The changes in preoperative and postoperative IOP.

	n = 171
Pre-op IOP, mean ± SD (range)	16.3 ± 2.4 (12–20)
Post-op IOP, mean ± SD (range)	14.4 ± 3.3 (8–18)
*p* value *	<0.001 *
Absolute IOP change, mean ± SD (range)	−3.0 ± 2.9 (−12.0–1.0)
% IOP change, mean ± SD (range)	−15.4 ± 18.7 (−50.0–7.1)

IOP: intraocular pressure, SD: standard deviation. * Wilcoxon signed-rank test.

**Table 3:** Correlation analyses for predictors of percent IOP change after cataract surgery.

	r Value	*p* Value *
Pre-op IOP	0.482	<0.001
ACD	−0.307	0.034
LT	0.383	0.026
AL	0.05	0.52
CCT	0.01	0.9

IOP: intraocular pressure, ACD: anterior chamber depth, LT: lens thickness, AL: axial length, CCT: central corneal thickness. * Pearson correlation test.

**Table 4:** Regression analysis of the association between preoperative parameters and changes in IOP percentage.

Predictors	Coefficient (95% Cl)	*p* Value *
Preoperative IOP	0.354 (0.243 to 0.465)	<0.001
ACD	−0.853 (−1.492 to −0.214)	0.009
LT	1.48 (0.7 to 2.26)	<0.001

IOP: intraocular pressure, ACD: anterior chamber depth, LT: lens thickness. * Multivariate regression analysis.

## Data Availability

The original contributions presented in the study are included in the article; further inquiries can be directed to the corresponding authors.
